# Label-free classification of breast cancer subtypes in ex vivo human tissues using Raman spectroscopy and machine learning

**DOI:** 10.1038/s41598-026-54071-5

**Published:** 2026-06-11

**Authors:** Ahmed Ezzat, Zhiyu Zhu, Alfie Roddan, Anna Silvanto, Yingwei Hou, Nilanjan Mandal, Jiacheng Xu, Ziqi Zhang, Mengyi Zhou, Ara Darzi, Simon Dryden, Daniel R. Leff, Alex J. Thompson

**Affiliations:** 1 Hamlyn Centre, Institute of Global Health Innovation, Imperial College London, London, SW7 2AZ UK; 2 Department of Surgery and Cancer, Imperial College London, London, SW7 2AZ UK

**Keywords:** Raman spectroscopy, Breast cancer, Machine learning, Subtype classification, Biomarkers, Cancer, Medical research, Oncology

## Abstract

Breast conserving surgery (BCS) aims to excise breast tumors whilst preserving breast-related quality of life, but is complicated by the challenge of accurately identifying the margin between healthy and cancerous tissue. Raman spectroscopy (RS) has been shown to distinguish between normal breast tissue and breast cancer. Thus, this study aimed to further evaluate the diagnostic performance of RS in ex vivo breast tissue subtype classification via investigation of signals from healthy tissues and three breast cancer subtypes (invasive ductal carcinoma, IDC; invasive lobular carcinoma, ILC; and ductal carcinoma in situ, DCIS). A total of 80 tissue samples (46 normal and 34 cancerous) from 71 individuals were measured using a confocal Raman microscope. Spectral signatures were investigated, and supervised classification was performed for both two-class (healthy vs. cancer) and four-class (healthy vs. IDC vs. ILC vs. DCIS) classification tasks. RS successfully differentiated cancerous from normal breast tissue (97.84% sensitivity, 97.18% specificity). For four-class classification, RS achieved in-class sensitivity ranging from 83 to 96% and specificity from 93 to 99%. These findings demonstrate that RS can accurately distinguish normal from cancerous tissue and capture clinically relevant differences among histological subtypes, including invasive and pre-invasive disease, supporting its promise for intraoperative tissue characterization during BCS.

**Supplementary Information:**

The online version contains supplementary material available at 10.1038/s41598-026-54071-5.

## Introduction

Breast conserving surgery (BCS) aims to remove malignant (cancerous) tissue while preserving as much healthy breast tissue as possible to maintain breast-related quality of life^1^. However, the inability to detect microscopic disease intraoperatively means that it is challenging to accurately identify the “margin,” which describes the distance between healthy and cancerous tissue. This results in a high reoperation rate, with nearly 20% of patients requiring re-excision following post-operative detection of “positive margins” – i.e. where, upon histological analysis, the tumor was found to be resected with an insufficient margin of surrounding healthy tissue^2^. Re-operative intervention contributes to physical, cosmetic, and psychosocial burdens for patients, along with significant financial implications. In the United Kingdom (UK), the National Health Service (NHS) spends over £23 million on more than 11,000 reoperations^3,4^.

Current intraoperative margin assessment tools, such as intraoperative radiography, offer limited sensitivity (41–50%)^5,6^. Therefore, more accurate and real-time techniques are urgently needed. Raman spectroscopy (RS), a label-free optical method based on inelastic scattering of monochromatic light, has shown promise in distinguishing tissue types by their biochemical composition (particularly proteins, lipids, and water content)^7^. Within the field of breast surgery, RS has been shown to accurately differentiate between healthy and invasive breast tissues with sensitivity and specificity of over 90%^8,9^.

Despite the substantial body of evidence supporting RS, most studies reported to date have focused primarily on two-class classification (between healthy and cancerous tissue). Importantly, however, clinical guidelines require different margin widths for different pre-invasive (B5a) or invasive (B5b) breast cancers^10–14^. For example, the recommended margin for ductal carcinoma in situ (DCIS) is 2 mm. Conversely, for invasive tumors such as invasive lobular carcinoma (ILC) and invasive ductal carcinoma (IDC), a margin of either “no ink on tumor” (i.e. a margin of > 0 mm) or at least 1 mm is considered adequate^10–14^. This means that an ideal intraoperative tool for breast cancer margin assessment would not only accurately differentiate between healthy and cancerous tissue but also between different breast cancer subtypes. To bridge this gap, this study aimed to evaluate the diagnostic performance of RS in ex vivo breast tissue differentiation for a diversity of histological breast cancer subtypes. Within our results, RS combined with machine learning enabled discrimination between cancerous and healthy breast tissues and provided proof-of-concept evidence for differentiating breast cancer subtypes, including IDC, ILC, and DCIS.

## Methodology

### Ex vivo human breast tissue sample collection and RS acquisition

To investigate RS-based identification of breast cancer subtypes, spectra were obtained from ex vivo human breast tissue samples using a confocal Raman microscope (DXR2xi, ThermoFisher Scientific). Ethical approval was provided by the Imperial College Healthcare Tissue Bank (ICHTB), with tissue specimens collected from consenting donors through ICHTB and Breast Cancer Now (registered charity no. 1160558). The study included a total of 80 frozen tissue samples from 71 individuals, comprising 46 histologically normal breast tissues and 34 cancerous breast tumors representing three distinct subtypes: 14 DCIS, 9 ILC, and 11 IDC. 9 patients contributed both normal and cancerous tissue samples. As these tissues exhibited histological differences, the corresponding spectra were processed according to their histological labels rather than being treated as paired samples from the same donor in subsequent classification analyses. Each sample was indexed using an anonymised patient-based identifier (Patient ID), such that spectra originating from the same donor shared the same ID. The thickness of tissue samples ranged from approximately 0.5–5 mm.

Prior to spectral acquisition, all samples were thawed. Raman measurements were performed using 785 nm laser excitation (2 mW at the sample) across a spectral range of 500–3000 cm^−1^, with an acquisition time of 0.2 s per spectrum. For all measurements, the Raman laser was manually focused onto the surface of the sample, meaning that spectra were collected from the most superficial tissue regions (i.e. <100 μm penetration depth). For each tumor sample, spectra were collected from 3 to 6 spatially distinct locations to account for intra-sample heterogeneity, while a single measurement point was selected for each normal tissue sample. At each measurement point, 2 to 3 spectra were acquired, resulting in a total of 641 Raman spectra used for subsequent analysis (177 from normal tissues and 464 from tumor samples: 221 from DCIS, 129 from ILC, and 114 from IDC).

### Histopathological validation

After Raman measurements, to enable validation against the diagnostic gold standard of histopathology, all samples were fixed, sectioned and then stained with Hematoxylin & Eosin (H&E) before being imaged under a microscope by a trained pathologist. This meant that samples were unstained at the point of Raman measurements. This histopathological assessment provided a “gold standard” diagnostic label for each tissue sample, which was used in all further analysis (e.g. when grouping samples/spectra according to subtype, when performing machine learning analysis, etc.). Importantly, however, due to the sample processing required for histopathology (sectioning, staining, etc.) and the inherent heterogeneity of samples, there was nonetheless some uncertainty in the histopathological diagnoses for each measured region (i.e. as each sample was categorized as a single tissue type via histology but the bulk tissues exhibited at least some degree of heterogeneity). In addition, normal tissue samples consisted of an approximately even mix of fibrous, adipose, and fibroadipose tissues. In the analysis presented here, all normal tissues were grouped into a single class to focus the investigation on identification and classification of cancer samples and cancer subtypes.

### RS data pre-processing procedure

To ensure RS data were suitable for use in downstream analysis and classification algorithms, a four-step pre-processing pipeline was designed and implemented using MATLAB. This included fingerprint region selection, smoothing, baseline correction, and normalization.

#### Selection of molecularly informative Raman region

The original dataset covers the spectral range of 500–3000 cm^−1^, including both the principal characteristic peaks of breast tissue and several regions lacking distinct features. Although more comprehensive information could potentially be obtained by using the full spectrum range, it also includes noisy regions, which increase the risk of misclassification^15^. Hence, the 800–2000 cm^−1^ spectral range was chosen as the Region of Interest (RoI) for subsequent analysis. This range covers the most informative fingerprint region, rich in characteristic peaks of biomolecules (proteins, lipids, nucleic acids, etc.)^16^ for which tissue concentrations are known to change in response to pathological changes at the molecular level^9^.

#### Smoothing technique

To suppress high-frequency noise, a Savitzky-Golay (SG) filter was used to smooth the spectra. The principle of this method is to fit a low order polynomial by least squares within a local window and replace the original points with the fitted values^17^.

Compared with simpler approaches such as median filtering, SG filtering more effectively preserves spectral peak shape, position, and intensity. In addition, although more complex methods (e.g. wavelet thresholding denoising) can perform better than SG filtering in extreme noise or strong fluorescent background scenarios, cosmic noise and strong fluorescence interference were not observed in this dataset meaning that SG filtering was well suited to the collected spectra. The filter parameters used in this study were a window size of 11 and a third-order polynomial, which balanced noise suppression with feature preservation.

#### Iterative polynomial baseline correction

For each spectrum, baseline correction was performed to eliminate non-characteristic signal interferences (e.g., fluorescence background, instrument drift, etc.). This was accomplished using an iterative polynomial fitting approach, as described in Ref^18^. This approach enabled baseline correction to be performed adaptively for different samples and measurement conditions, without requiring separate acquisition of a fluorescence background signal for subtraction.

Each Raman spectrum was initially fitted using a high-order polynomial to estimate a preliminary baseline. At iteration * k*, the spectrum data used for baseline fitting is denoted as $\:{y}_{i}^{\left(k\right)}$, where the first iteration uses the original spectrum, i.e., $\:{y}_{i}^{\left(1\right)}={y}_{i}$. The residual between the input data and the estimated baseline is calculated as $\:{r}_{i}^{\left(k\right)}={y}_{i}^{\left(k\right)}-{b}_{i}^{\left(k\right)}$, where $\:{b}_{i}^{\left(k\right)}$ represents the baseline estimated by the *k*^*th*^ polynomial fit. The corresponding residual standard deviation was computed as.

1 $$\:{{\upsigma\:}}^{\left(\mathrm{k}\right)}=\sqrt{\frac{1}{\mathrm{N}}\sum\:_{\mathrm{i}=1}^{\mathrm{N}}{\left({\mathrm{r}}_{\mathrm{i}}^{\left(\mathrm{k}\right)}\right)}^{2}\:\:.}$$

To improve baseline accuracy, an updated version of the spectrum is generated for the next iteration by retaining low intensity points below the estimated baseline and replacing points above the baseline with the baseline values calculated in the previous iteration. This update rule is explained mathematically as:

2 $$\:{\mathrm{y}}_{i}^{\left(\mathrm{k}\right)}=\left\{\begin{array}{c}{\:\:\:\:\:y}_{i}^{\left(\mathrm{k}\right)}\:\:\:\:\:\:\:\:if\:{y}_{i}^{\left(\mathrm{k}\right)}<{\mathrm{b}}_{i}^{\left(\mathrm{k}\right)}\:\\\:{\mathrm{b}}_{i}^{\left(\mathrm{k}\right)}\:\:\:\:\:\:\:\:\:\:otherwise\end{array}\right.$$

The iteration process continued until the relative change in standard deviation between successive iterations fell below a predefined threshold ($\:{\upepsilon\:}$) indicating convergence:

3 $$\:\varDelta\:{\upsigma\:}=\frac{|{{\upsigma\:}}^{\left(\mathrm{k}\right)}-{{\upsigma\:}}^{(\mathrm{k}-1)}|}{{{\upsigma\:}}^{(\mathrm{k}-1)}}<{\upepsilon\:}.$$

Once convergence was achieved, the final estimated baseline was subtracted from the original spectrum to obtain the baseline-corrected spectrum. In this work, the baseline was modeled using a fifth-order polynomial, and convergence was defined as a 5% relative change in residual standard deviation between successive iterations (i.e. ε = 0.05).

#### Normalization

To account for variation in signal intensity across samples and measurements (e.g. due to variations in laser power, sample structure, detector sensitivity, etc.), Z-score normalization (standard score transformation) was applied to all spectra. This method standardizes the intensity values of each spectrum by transforming them into a distribution with a mean of zero and a standard deviation of one, effectively eliminating scale differences among features^19^.

This normalization also ensures the data centering required for subsequent Principal Component Analysis (PCA), preventing features with larger variances from disproportionately influencing classification models.

### RS-based tissue classification

Aiming to support both preoperative diagnosis and intraoperative resection guidance, a spectral classification pipeline was developed based on Raman spectroscopy and machine learning techniques.

After pre-processing the raw data into a standardized format, dimensionality reduction and supervised learning techniques were applied to extract diagnostic features and build classifiers. PCA was used to reduce feature dimensionality, followed by classification using Linear Discriminant Analysis (LDA) and Support Vector Machines (SVM) respectively. Given the limited dataset, these computationally efficient classifiers were chosen to promote generalizability and mitigate overfitting. All processing was performed using MATLAB.

Using both LDA and SVM, both two-class and four-class classifications models were developed. Two-class models aimed to perform binary classification of cancerous vs. normal samples (with the cancer class representing both invasive and pre-invasive disease – i.e. combining all DCIS, IDC and ILC samples). Four-class models aimed to achieve classification of tissue subtype (i.e. normal vs. DCIS vs. IDC vs. ILC).

#### PCA dimensionality reduction

To implement PCA, the normalized spectral data were first used to compute the covariance matrix. Eigenvalue decomposition was then performed to extract Principal Components (PCs), which were ranked based on the amount of variance they explained. The top PCs were selected to form a reduced feature set for subsequent classification, aiming to preserve critical spectral information while minimizing noise and redundancy.

#### LDA classification

LDA is a classical supervised classification method widely used in RS analysis^20,21^. Its primary goal is to identify a projection direction that maximizes the separation between classes while minimizing variation within each class^21^.

However, under conditions of high dimensionality and small samples, direct application of LDA may lead to a singular within-class scattering matrix, causing the training process to fail. Therefore, PCA was first used for dimensionality reduction, and LDA was then applied to the extracted PC scores to build a classifier. This approach ensured computational stability and enhanced the robustness of the subsequent LDA classification.

#### SVM classification

SVM is a supervised classification algorithm based on the principle of margin maximization, aiming to identify an optimal hyperplane for separating data^22^. In contrast to LDA, which emphasizes global discriminative structure, SVM offers greater flexibility in constructing decision boundaries within the feature space. It is particularly suitable for processing data with complex or nonlinear distributions. Similarly, PCA was first applied for dimensionality reduction, and the resulting PC scores extracted from each spectrum were then used to train the SVM classifier. To perform multi-class classification, SVM was implemented using a radial basis function (RBF) kernel combined with a one-vs-one error-correcting output codes (ECOC) strategy, as provided by MATLAB “fitcecoc” function. This configuration enables the model to handle both nonlinearity in the data and classification across multiple classes. In all SVM models, the kernel scale (γ) was set to the MATLAB “auto” option (which automatically estimates the kernel width from the training data) and the regularization strength (C) was fixed at 10. A regularization strength of C = 10 was chosen following a simple manual parameter sweep, which demonstrated that performance increased as C increased from 0.1 to 10 but then stayed approximately constant as C increased to 100.

In this study, LDA and SVM were chosen as complementary classification models due to their distinct strengths. LDA, as a linear discrimination method, is efficient and robust for linearly separable data. In contrast, SVM could be extended to nonlinear feature spaces via kernel functions, enabling it to capture more complex class boundaries. The combination of both models provides a comprehensive evaluation of RS classification performance under varying distributional scenarios.

#### Validation model

To assess the accuracy and generalization ability of the classification models, k-fold cross-validation was used (with both k = 10 and k = 20 tested). To avoid data leakage due to multiple spectra originating from the same patient (including those from different tissue samples and histological regions), we ensured that no patient contributed spectra to both the training and test sets within a given fold. Specifically, after initial folding assignment, any spectra from patients appearing in the training set were removed from the corresponding test folds. Additionally, to determine the optimal number of PCs, cross-validation was repeated using different numbers of PCs in the classification models (ranging from 1 to 300 PCs). Classification accuracy was calculated for all PC combinations. Results were then compared between the highest accuracy and those obtained at the number of PCs explaining 90% and 95% of the variance, to assess performance and examine the potential risk of overfitting (for both LDA and SVM). Nested cross-validation was not used due to the limited sample size, which would further reduce training data per fold.

## Results

### RS profiles of normal and breast cancer tissue samples

**Fig. 1:**
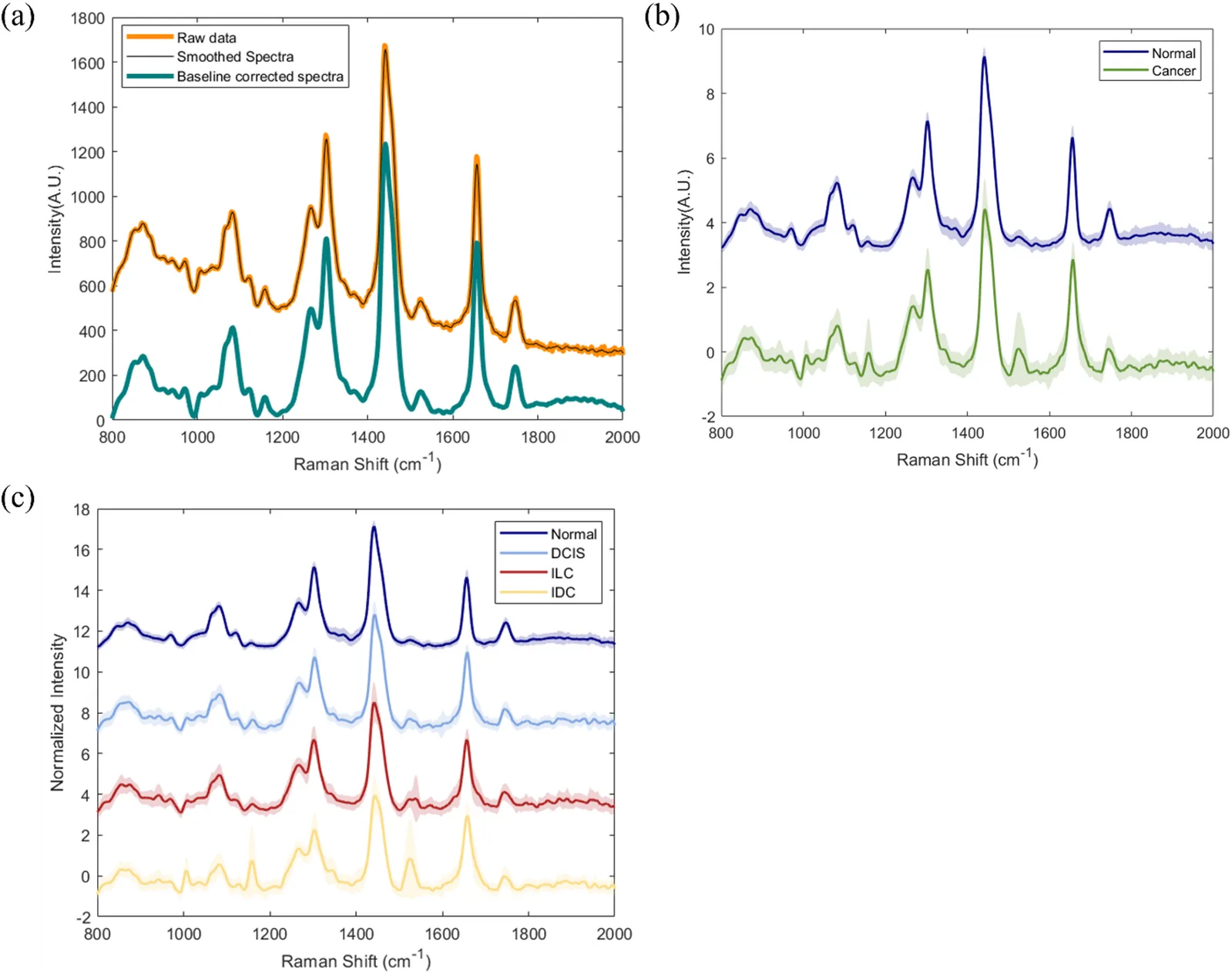
Breast tissue Raman spectra. (**a**) Raman spectra pre-processing steps (demonstrated on an example spectrum). (**b**) Mean normalized spectra of cancer (i.e. combing all IDC, ILC and DCIS spectra) and normal samples (with standard deviations). (**c**) Stacked normalized spectra (mean ± standard deviation) of all four classes for comparison.

Figure 1 (a) first illustrates a typical spectral profile at each stage of pre-processing. The orange curve represents raw data, where fluctuations are especially evident in the lower intensity region. After applying SG filtering (window length: 11, polynomial order: 3), the black curve demonstrates spectral smoothing (reduction in high frequency noise) while retaining the original shape and peak positions. As further validated in Fig. S1, the smoothing process also effectively removed false (misidentified) peaks, while preserving true spectral features.

Baseline correction was subsequently performed using a fifth-order polynomial, with an iterative threshold set to 5% for convergence. In Fig. 1 (a), the green curve represents the data after baseline correction, in which the broad downward trend in the raw data has been eliminated after correction. Peak positions remained unchanged after baseline subtraction, confirming that the spectral shape was not distorted and only the baseline was eliminated. This is further supported in Fig. S1, which shows consistent peak locations before and after baseline correction.

Figure 1(b-c) display the normalized Raman spectra for all sample groups (scaled to a range of − 1 to 6 via Z-score normalization), highlighting their average spectral profiles and inter-sample variability. Each curve represents the mean spectrum, and the shaded ribbons denote the standard deviation.

Good signal-to-noise ratio was observed in the spectra obtained from all tissue types, with a degree of variability that was attributed to tissue heterogeneity (Fig. 1(b-c); also see discussion of histopathological validation in the Methodology section. Among the groups, normal spectra exhibited the highest consistency, characterized by the lowest spectral variability. In contrast, cancerous samples – particularly IDC – displayed greater variation, suggesting more substantial molecular diversity and biological heterogeneity within these samples. Importantly, qualitative differences are observable between groups (both between cancer and normal samples, and between subtypes) in terms of both the presence and relative intensities of Raman peaks (Fig. 1(b-c)).

### Interpretation of RS differences between breast tissue types

**Fig. 2:**
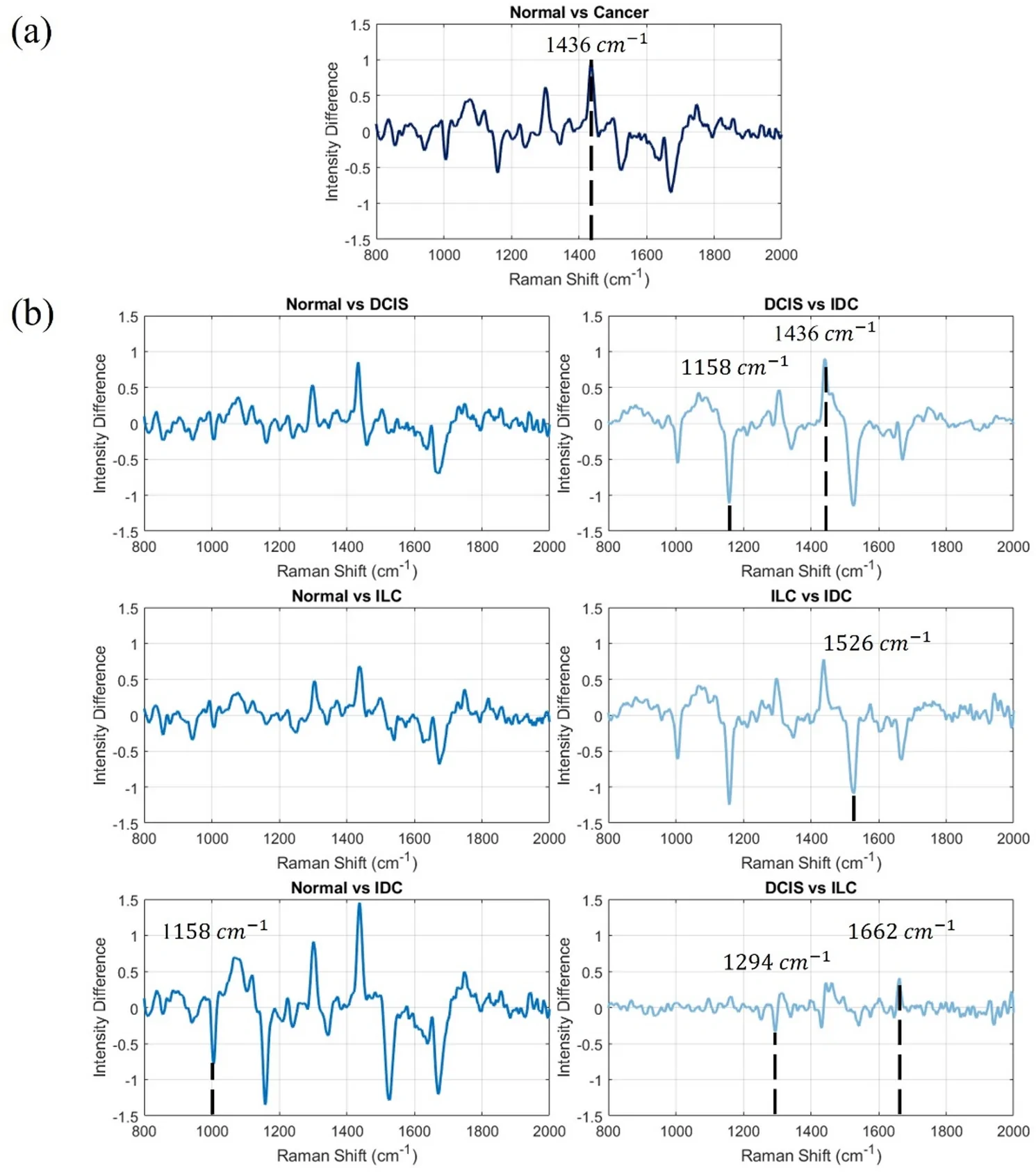
Difference spectra highlighting spectral variations that contribute to classification. (**a**) Difference spectrum between Normal and Cancer samples. (**b**) Pairwise difference spectra among all four tissue classes (normal, DCIS, ILC, and IDC). Dotted black lines indicate example spectral peaks where differences are observed between classes.

To further explore spectral differences between sample groups, Fig. 2 displays the difference spectra obtained through pairwise subtraction of the average spectra between groups. Specifically, the order of subtraction follows the labels in each panel of Fig. 2 (e.g., “Normal vs. Cancer” in Fig. 2(a) corresponds to $\:{I}_{\mathrm{normal}}\left(\nu\:\right)-{I}_{\mathrm{cancer}}\left(\nu\:\right)$, where *ν* represents Raman shift). Thus, positive values indicate Raman shifts at which the first group has higher mean intensity than the second group, whereas negative values indicate wavenumbers where the first group exhibits lower mean intensity. This method highlights a number of distinct spectral differences between groups at specific Raman shifts.

For example, in Fig. 2(a), the Raman peak at 1436 $\:\mathrm{c}{\mathrm{m}}^{-1}$ (associated with lipid-related CH₂ bending/stretching/scissoring molecular vibrations)^2^ shows higher intensity in normal samples compared to cancerous tissues. Similar lipid-related differences are evident in the left column of Fig. 2(b), which shows the differential spectra for comparison of each cancer subtype against normal tissues. This demonstrates that the lipid-related peak at 1436 cm^-1^ is suppressed (relative to normal tissue) in all of the cancer subtypes investigated here (DCIS, IDC, ILC).

While the subtype difference spectra share overall similarities, they also exhibit subtype-distinct features. For instance, a clear difference is observed at 1158 $\:\mathrm{c}{\mathrm{m}}^{-1}$ (corresponding to the C–C stretching vibration of β-carotene)^16^ in the comparison between normal and IDC tissues but is absent in the comparisons between normal tissue and DCIS or ILC. Interestingly, this observation contrasts with previous work by Redd et al., where the 1158 cm^-1^ peak was reported as having higher intensity in normal tissues than in breast cancer tissues (albeit with different experimental parameters to those used here)^23^.

 Finally, the right column of Fig. 2(b) illustrates the spectral differences among cancer subtypes. Strong spectral separations are observed between IDC and the other two tissue classes (DCIS and ILC; Fig. 2(b), top-right and middle-right panels). For example, IDC shows higher intensities at 1158 and 1526 cm^−1^, but a lower intensity at 1436 cm^−1^ compared to both DCIS and ILC. In contrast, DCIS and ILC exhibit smaller (although still visually distinguishable) spectral differences (Fig. 2(b), bottom-right panel). For example, differences between DCIS and ILC are observed at 1294 and 1662 cm^−1^, with ILC exhibiting stronger intensity at 1294 cm^−1^ and DCIS showing higher signal at 1662 cm^−1^. These results highlight both the potential and the challenges of RS-based tissue classification among breast cancer subtypes. A detailed summary of peak assignments and associated biochemical components is provided in Table S1.

### Feature extraction

**Fig. 3:**
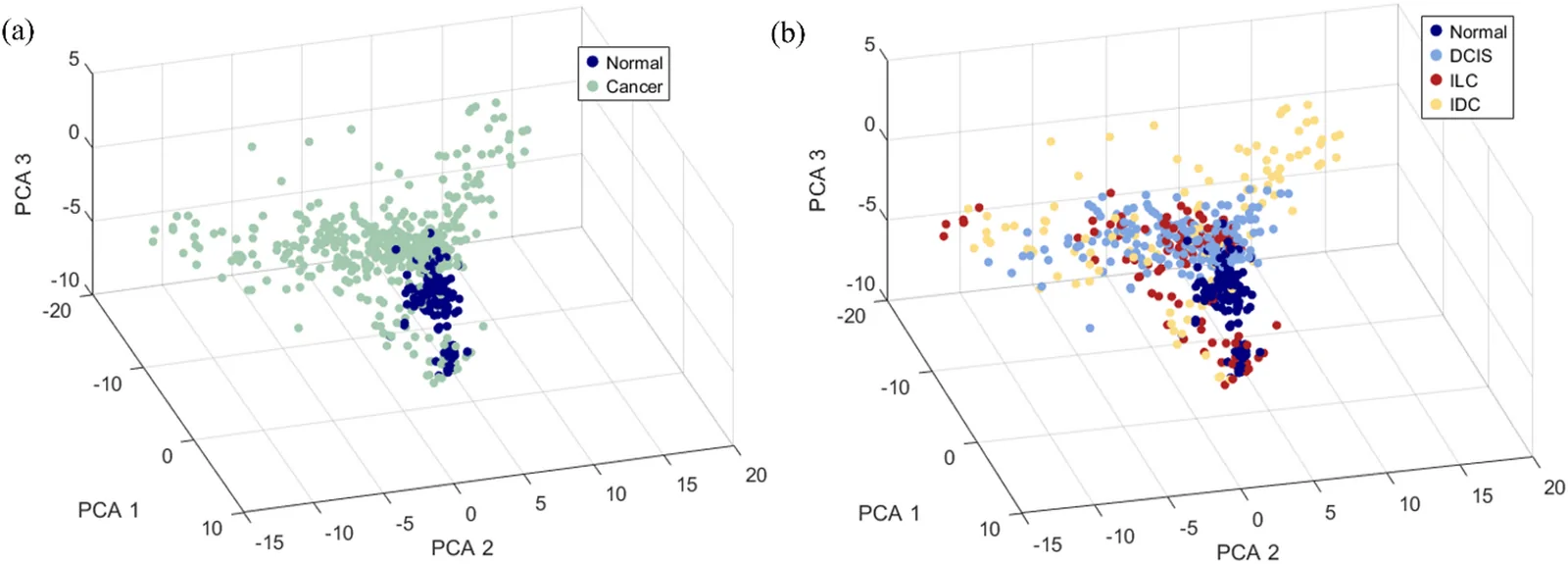
Three-dimensional PCA plots, where the first three principal components are used to visualize clustering patterns among tissue types. (**a**) 3D PCA plot showing the distribution of normal and cancerous samples. (**b**) 3D PCA plot showing the distribution of all four tissue classes: normal, DCIS, ILC, and IDC.

Figure 3 shows three-dimensional PCA visualizations of the RS data, which can be used to evaluate the distribution of different tissue types in the PCA feature space. Figure S2 provides additional scatter plots for PC1 vs. PC2, PC2 vs. PC3, and PC1 vs. PC3. The results are consistent with the mean spectra and difference spectra, showing a tendency toward separation between cancerous and normal groups and revealing a greater degree of variability in the cancerous samples (Fig. 3(a)). Among cancer subtypes (Fig. 3(b)), IDC samples are more widely distributed, exhibiting the greatest variability. In addition, DCIS and ILC groups show partial overlaps, further confirming their spectral similarity.

Overall, PCA effectively demonstrates the separability of the dataset by clustering similar tissue types and separating distinct ones. While considerable overlap is observed in the 3-component PCA plot between certain subtypes (such as DCIS and ILC), the dimensionality reduction nonetheless facilitates a degree of visual differentiation between groups (see Fig. 3 and Fig. S2), indicating potential for RS-based classification.

### Classification results

#### LDA classification

**Fig. 4:**
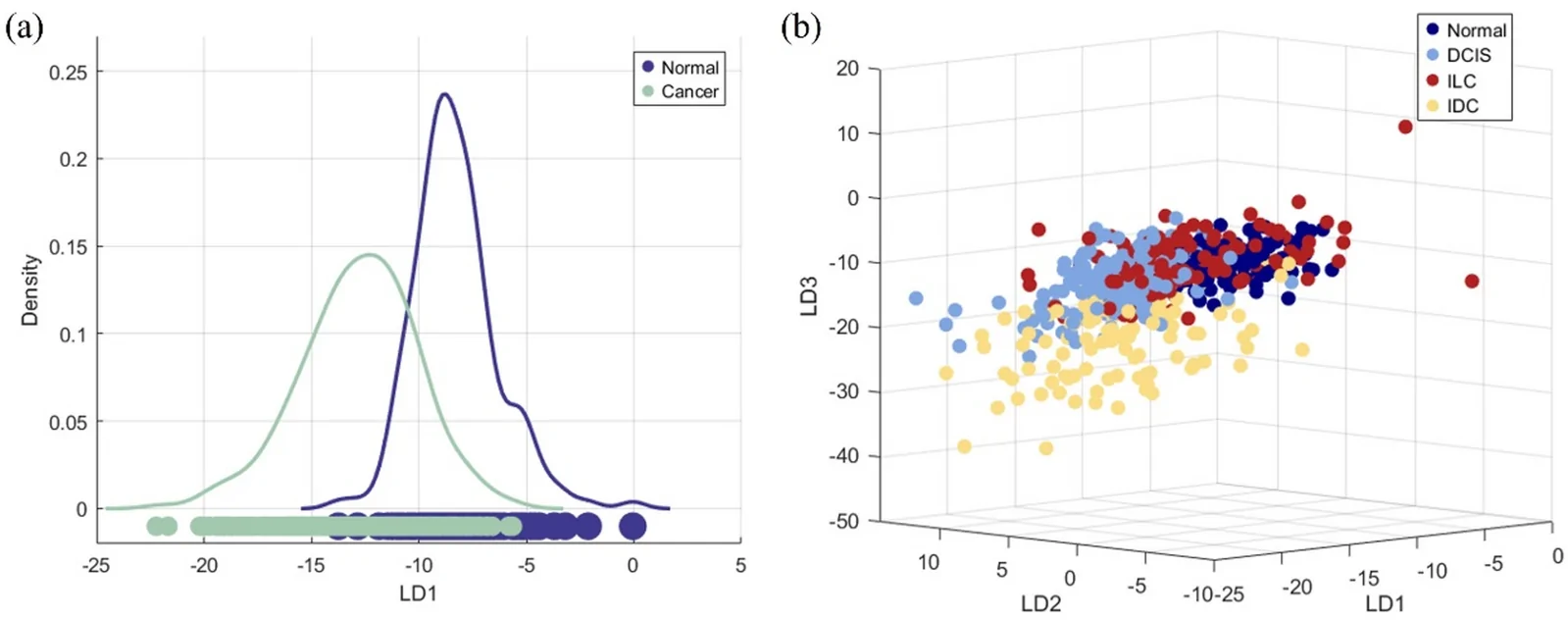
LDA-based discrimination of tissue classes. (**a**) LD1 density distribution for Normal and Cancer groups. (**b**) Three-dimensional LDA plot showing the distribution of normal, DCIS, ILC, and IDC samples along the three linear discriminants (LDs). Graphs were generated using the LDA models found to exhibit optimal performance (i.e. using 182 PCs for two-class classification, 178 PCs for four-class classification).

After PCA dimensionality reduction, the data was modeled using LDA for classification. Figure 4 shows the LDA discrimination observed in the two- and four-class classification tasks respectively. In two-class classification (normal vs. cancer), although LDA achieves a certain level of separation between cancerous and normal samples along the first Linear Discriminant (LD1), overlapping regions remain (Fig. 4(a)). The distribution curves indicate that samples from the two classes are intermixed near the decision boundary along the LD1 axis, despite differences in their peak positions. This overlap limits the ability of the model to fully differentiate the two classes.

Similar findings are observed in the four-class classification (i.e. normal vs. DCIS vs. ILC vs. IDC). As shown in Fig. 4(b), while IDC appears well separated from the other tissue classes, the remaining three classes exhibit overlap in the three-dimensional discriminant space. This observation is further supported by Fig. S3, where two-dimensional LD projections reveal the challenge of distinguishing spectrally similar subtypes using LDA.

**Fig. 5:**
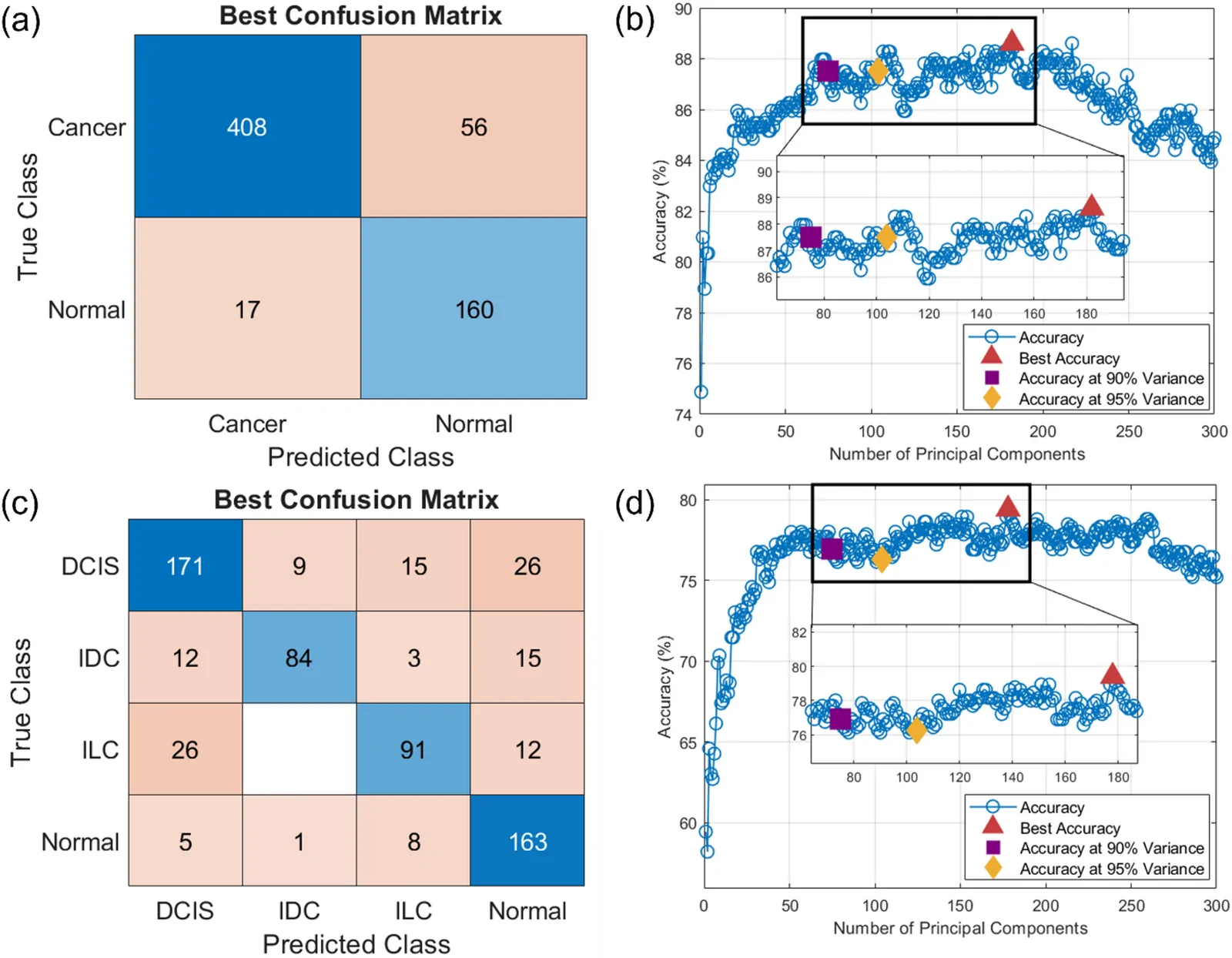
LDA classification results with 20-fold cross-validation. (**a**) Confusion matrix for binary classification (cancer vs. normal). (**b**) Classification accuracy as a function of number of PCs included in classification model for the binary task. Three highlighted markers indicate best accuracy (red triangle, 182 PCs), accuracy at the number of PCs explaining 90% of the total variance (purple square, 75 PCs), and accuracy at the number of PCs explaining 95% of total variance (yellow diamond, 104 PCs). Inset plot shows detail of graph around the highlighted accuracies. (**c**) Confusion matrix for four-class classification. (**d**) Classification accuracy as a function of number of PCs included in classification model for the four-class task. Three highlighted markers indicate best accuracy (red triangle, 178 PCs), accuracy at 90% explained variance (purple square, 75 PCs), and accuracy at 95% explained variance (yellow diamond, 104 PCs). Inset plot shows detail of graph around the highlighted accuracies. Confusion matrices were calculated based on optimal model performance (using 182 PCs for two-class classification, 178 PCs for four-class classification).

 Despite these observations, LDA was nonetheless able to provide good classification accuracy. The confusion matrices in Fig. 5(a) and (c) – corresponding to two- and four-class classification tasks respectively (with 20-fold cross-validation; results obtained with 10-fold cross-validation are also presented in Fig. S4 and Table S2) – further illustrate the degree of overlap in the LDA transformation space (through the misclassifications observed in each class) but also demonstrate that the majority of samples (i.e. approx. 70–90%) are classified correctly in each class. Table 1 summarizes the detailed classification metrics obtained for each class (for both two- and four-class classification) with 20-fold cross-validation. The two-class model demonstrated strong performance with high sensitivity (87.93%), specificity (90.40%) and accuracy (88.16%), indicating reliable differentiation between cancerous and normal tissues.

In the four-class classification task, the highest specificity (98.10%) was observed in IDC vs. non-IDC, indicating that IDC is highly distinguishable from other classes. Conversely, ILC vs. non-ILC classification had the lowest sensitivity (70.54%), highlighting the challenge of accurately recognizing ILC. Nonetheless, the overall classification accuracy was 79.4% in the four-class task, demonstrating good discrimination between classes albeit with an appreciable number of misclassifications.

**Table 1:** LDA classification performance metrics for two- and four-class classification tasks, including sensitivity, specificity, precision, overall accuracy, and accuracy at the number of PCs explaining 90% and 95% of total variance (based on 20-fold cross-validation). Metrics (except for 90/95% explained variance accuracy) were calculated based on optimal model performance (using 182 PCs for two-class classification, 178 PCs for four-class classification).

Classification task	Classes	Sensitivity	Specificity	Precision	Total accuracy	90/95% Explained variance accuracy
Two-class	Cancer vs. normal	87.93%	90.40%	96.00%	88.16%	87.50%/ 87.50%
Four-class	DCIS vs. non-DCIS	77.38%	89.76%	79.91%	79.4%	76.92%/ 76.29%
	IDC vs. non-IDC	73.68%	98.10%	89.36%		
	ILC vs. non- ILC	70.54%	94.92%	77.78%		
	Cancer vs. normal	92.09%	88.58%	75.46%		

Figure 5(b) and (d) show the trends in overall accuracy (for two- and four-class classification respectively) as the number of PCs included in the classification models increased from 1 to 300. As the number of PCs rises, accuracy increases until a maximum is reached (at 182 PCs for two-class classification, at 178 PCs for four-class). Increasing the number of PCs beyond that point then results in a plateau and finally reduction in performance (e.g. due to the inclusion of noise and/or redundant information in the classification models).

For both two- and four- class classification, the PC combination yielding the best performance included a greater number of PCs than the 95% explained variance threshold, with accuracy approximately 1–3% higher than that observed at the 90% and 95% explained variance levels (see coloured markers in Fig. 5(b) and (d)). Importantly, accuracy within the optimal performance regions (see insets of Fig. 5(b) and (d)) varied by only approximately $\:\pm\:$2%, demonstrating that there was limited or no impact of overfitting at the peak performance point. Thus, the values presented in Fig. 5(a) and (c) and Table 1 represent those obtained at the maximum performance level (i.e. with 182 PCs for two-class classification, with 178 PCs for four-class classification).

#### SVM classification

**Fig. 6:**
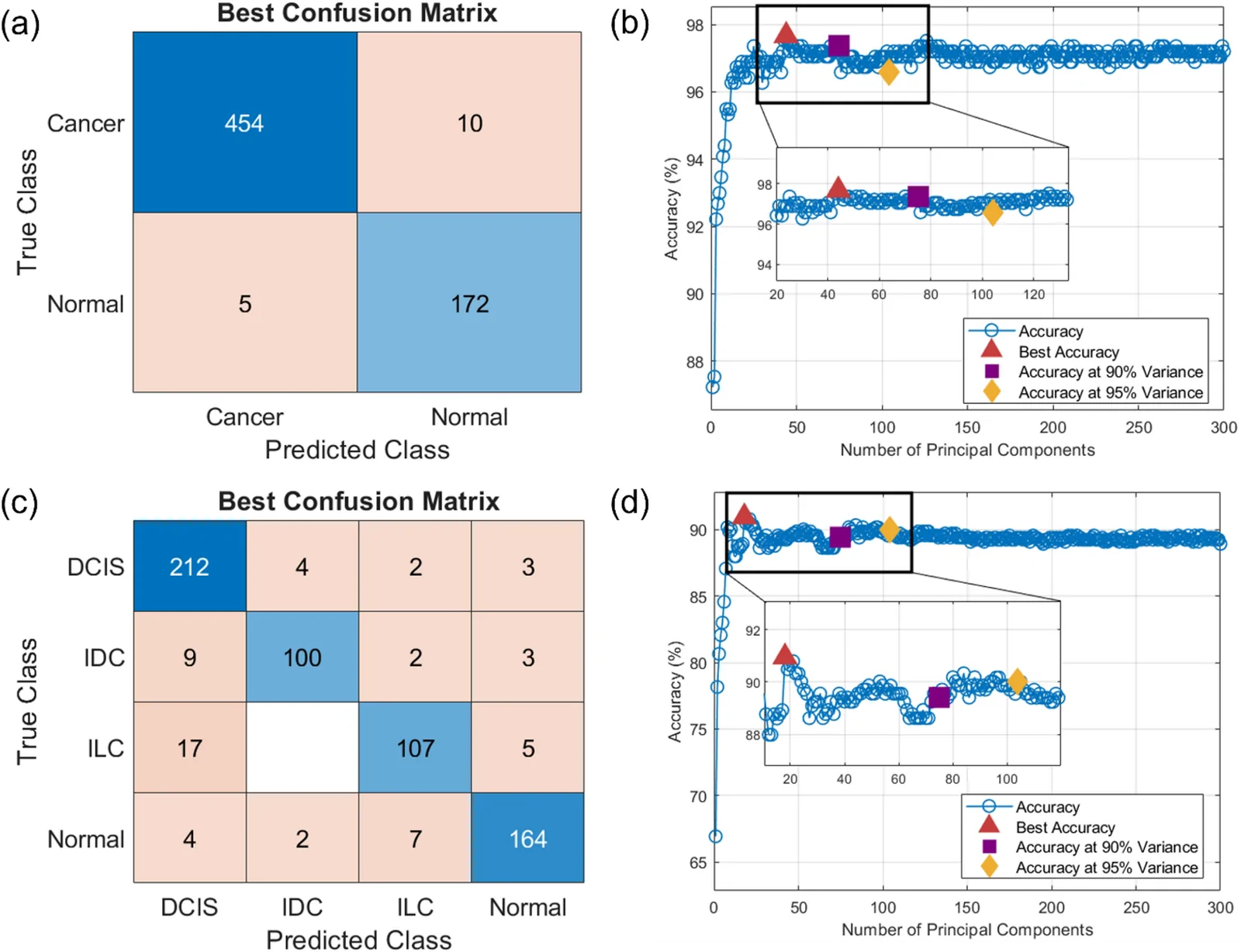
SVM classification results with 20-fold cross-validation. (**a**) Confusion matrix for binary classification (cancer vs. normal). (**b**) Classification accuracy as a function of number of PCs included in classification model for the binary task. Three highlighted markers indicate best accuracy (red triangle, 44 PCs), accuracy at the number of PCs explaining 90% of the total variance (purple square, 75 PCs), and accuracy at the number of PCs explaining 95% of total variance (yellow diamond, 104 PCs). Inset plot shows detail of graph around the highlighted accuracies. (**c**) Confusion matrix for four-class classification. (**d**) Classification accuracy as a function of number of PCs included in classification model for the four-class task. Three highlighted markers indicate best accuracy (red triangle, 18 PCs), accuracy at 90% explained variance (purple square, 75 PCs), and accuracy at 95% explained variance (yellow diamond, 104 PCs). Inset plot shows detail of graph around the highlighted accuracies. Confusion matrices were calculated based on optimal model performance (using 44 PCs for two-class classification, 18 PCs for four-class classification).

 Based on the same pre-processing and PCA dimensionality reduction, SVM was then also used for classification modeling. Table 2; Fig. 6 demonstrate the performance of SVM in two- and four-class classification tasks with 20-fold cross-validation. (Similar performance was observed using 10-fold cross-validation – see Fig. S5 and Table S3).

**Table 2:** SVM classification performance metrics for two- and four-class classification tasks, including sensitivity, specificity, precision, overall accuracy, and accuracy at the number of PCs explaining 90% and 95% of total variance (based on 20-fold cross-validation). Metrics (except for 90/95% explained variance accuracy) were calculated based on optimal model performance (using 44 PCs for two-class classification, 18 PCs for four-class classification).

Classification task	Classes	Sensitivity	Specificity	Precision	Total accuracy	90/95% Explained variance accuracy
Two-class	Cancer vs. normal	97.84%	97.18%	98.91%	97.67%	97.36%/ 96.57%
Four-class	DCIS vs. non-DCIS	95.93%	92.86%	87.60%	90.96%	89.40%/ 90.02%
	IDC vs. non-IDC	87.82%	98.86%	94.34%		
	ILC vs. non- ILC	82.95%	97.85%	90.68%		
	Cancer vs. normal	92.66%	97.63%	93.71%		

As shown in Fig. 6(a), the SVM model exhibits high accuracy classification in the two-class classification task. The overall classification accuracy reaches 97.67% (Table 2), considerably higher than the 88.16% overall accuracy achieved using LDA (Table 1). The sensitivity and specificity observed for two-class SVM classification both exceed 97%, indicating that the model correctly recognizes the two types of samples with a very low misclassification rate. Figure 6(b) shows that the SVM model reaches optimal performance when the number of principal components is 44, and the accuracy stabilizes at approx. 97% after the number of included PCs exceeds 100.

In the four-class task, all SVM metrics are considerably higher than those observed using LDA (compare Table 2 against Table 1), and Fig. 6(c) demonstrates that a high proportion of samples are correctly identified using SVM. Importantly, SVM exhibits improved classification performance (relative to LDA) in the ILC group, indicating that SVM is more adaptable than LDA in dealing with spectral feature overlap and boundary complexity problems. Figure 6(d) shows that the model accuracy is highest when using 18 PCs, with an overall accuracy of over 90%.

In both the two-class and four-class classification tasks, SVM achieved optimal performance with a relatively small number of PCs, below the threshold corresponding to 90% of explained variance (see coloured markers in Fig. 6(b) and (d)). This indicates that there was limited or no impact of overfitting. It also further highlights the ability of the SVM to capture discriminative spectral information in low-dimensional space while minimizing noise from higher-order PCs.

#### LDA and SVM comparison

To quantitatively compare the performance of the SVM and LDA models, each was run 5 times (k = 20), and the classification accuracy was recorded. Wilcoxon signed-rank tests were then performed to compare accuracy values across the SVM and LDA models. As illustrated in Fig. S6, both the two- and four-class classification tasks yielded statistically significant differences between the two models (*p* < 0.001). In both cases, SVM achieved consistently higher median classification accuracy than LDA, indicating a clear performance advantage. This demonstrates that the nonlinear modeling capabilities and feature adaptability of the SVM model make it particularly well suited for processing Raman spectral data with complex decision boundaries and class overlap features. These findings emphasize the advantages of incorporating nonlinear classifiers when dealing with highly heterogeneous biological samples, as they offer potential to improve classification accuracy and model robustness.

 Additionally, classification performance was also evaluated using 10-fold cross-validation with both LDA and SVM models. The corresponding confusion matrices and classification metrics are presented in Fig. S4&5 and Tables S2&3 (Supplementary Information), respectively. The 10-fold cross-validation results were consistent with those obtained using 20-fold cross-validation.

 Finally, to complement the four-class classification and provide a more clinically interpretable evaluation, we further performed binary comparisons of normal tissue against each cancer subtype (i.e., Normal vs. DCIS, Normal vs. ILC, and Normal vs. IDC) using both LDA and SVM (see Tables S4&5, Supplementary Information). These binary analyses provided similar accuracies to those observed in the four class classification tasks, thus further indicating the potential for subtype-level classification. In addition, the binary analysis results also further demonstrated that SVM provided improved classification accuracy over LDA.

## Discussion

In this study, we investigated the use of ex vivo RS for differentiating normal and malignant breast tissue and classifying histopathological breast cancer subtypes. RS demonstrated excellent diagnostic performance in the two-class classification task, achieving 97.84% sensitivity, 97.18% specificity, 98.91% precision, and an overall accuracy of 97.67%. These findings are consistent with prior studies and further validate the clinical potential of RS in breast cancer diagnostics^24–29^.

Early work demonstrated the feasibility of RS in breast cancer detection, reporting 83% specificity and 93% sensitivity on a small cohort of 22 patients^9^. More recently, studies involving larger datasets (e.g., > 500 samples) have reported RS-based sensitivities as high as 95%^30^. Our findings build upon this foundation by confirming the diagnostic accuracy of RS in an independent dataset and by extending the application beyond binary classification.

Spectral interpretation of the RS data showed key biochemical differences between normal and cancerous tissue. As reported previously, normal breast tissue exhibited higher lipid content, while malignant tissue showed increased levels of proteins and nucleic acids^16^. In our dataset, this was reflected by elevated intensity at 1436 cm^−1^ in normal samples (a lipid-related peak), and stronger signals at 1245 cm^−1^ (ceramide), 1090 cm^−1^ (proteins/nucleic acids), and 1583 cm^−1^ (phenylalanine) in cancerous tissue^16,31,32^.

Importantly, we extended the analysis beyond two-class classification by evaluating the performance of RS in a four-class classification task targeting healthy tissue and three breast cancer subtypes: DCIS, IDC, and ILC. To date, few studies have explored RS-based discrimination among these histopathological subtypes. Our analysis demonstrated > 82% sensitivity and specificity across all subtype classifications, with SVM-based models outperforming LDA in both binary and multi-class tasks.

This result is highly encouraging. Distinguishing DCIS from invasive subtypes is clinically important given differences in recommended margin width for DCIS versus invasive breast cancer. Accurate delineation of pre-invasive and invasive diseases at resection margins would help ensure adequate surgical margins, guide immediate further resection and reduce the risk of positive margins requiring re-excision. This clinical motivation is consistent with previous ex vivo breast tissue studies demonstrating the feasibility of Raman spectroscopy for surgical margin identification^33,34^. ILC also presents unique diagnostic challenges due to its diffuse growth pattern and frequent lack of radiological visibility, which make detection and intraoperative margin assessment more difficult^35,36^.

A recent study involving 804 patients used SVM to classify RS data obtained from blood serum, reporting around 94% accuracy^36^. However, that study did not use tissue samples and did not address multi-class tissue subtype classification. In contrast, our work focused on tissue-based spectral analysis and demonstrated strong performance in discriminating between DCIS, IDC, and ILC. Additionally, our findings are supported by previous work using mid-infrared spectroscopy, which showed promise in subtype differentiation, though without resolving individual invasive subtypes^37^.

Despite these promising and positive results, this study has several limitations. Most importantly, it is based on ex vivo tissue measurements using a benchtop Raman microscope, which may not reflect the conditions of intraoperative or in vivo applications. Intraoperative factors such as the presence of blood, tissue hydration levels, and perfusion may alter the Raman signal quality or introduce additional background noise, thereby affecting spectral interpretation and classification performance. Second, although Raman measurements were validated against histopathology, there were nonetheless uncertainties in the histopathological diagnoses of the exact tissue locations that were measured (due to sample heterogeneity and challenges in accurately mapping histopathology results from sectioned tissue back to locations of Raman measurements made on bulk tissue).

Furthermore, while this study serves as a proof-of-concept evaluation of the RS–machine learning pipeline on ex vivo tissues, it does not yet address the full spectrum of clinical boundary conditions relevant to margin assessment (e.g. no measurements were made on marginal tissues or tissues with mixed pathologies). Future work should therefore validate the approach in more clinically representative settings and samples, and investigate portable Raman implementations for real-time intraoperative margin evaluation. Notably, similar efforts have already been demonstrated in other cancer types. For example, fiber-optic Raman probes combined with machine-learning classifiers have been successfully applied for intraoperative detection of oral cancer margins, achieving 91% diagnostic accuracy^38^. In parallel, our group is currently developing a fibre-optic surface-enhanced Raman scattering (SERS) device designed for clinical applications in both cancer and infection diagnostics^39^. In vivo studies using such systems will be essential to evaluate the robustness of RS under physiological conditions and in heterogeneous clinical populations.

Finally, the relatively restricted sample size may limit statistical confidence in diagnostic performance, highlighting the need for larger studies to confirm these findings. Indeed, although no obvious signs of overfitting were identified in the current analyses, the possibility of overfitting cannot be entirely excluded (in part due to the limited sample size).

In conclusion, our study highlights the high diagnostic accuracy of RS in both two-class (healthy vs. cancer) and histological subtype classification of breast tissue. The strong performance of the SVM classifiers, consistent with existing literature, provides strong proof-of-concept that machine learning-enhanced RS can have significant potential for intraoperative guidance in BCS. Further external validation in larger, independent cohorts, together with clinically deployable RS instrumentation and workflows, will be the key steps toward routine surgical translation.

## Supplementary Information

Below is the link to the electronic supplementary material.

Supplementary Material 1

## Data Availability

The dataset generated and analyzed in this study, together with the MATLAB code used for spectral preprocessing, feature extraction, and machine-learning classification, has been deposited in Zenodo and is publicly available at DOI: 10.5281/zenodo.20042934.

## Declarations

### Approval for human experiments

Ethical approval for this study was provided by the Imperial College Healthcare Tissue Bank (ICHTB). Human tissue specimens were collected through ICHTB and Breast Cancer Now (registered charity no. 1160558), and informed consent was obtained from all subjects and/or their legal guardians prior to sample collection. All experiments were performed in accordance with Good Clinical Practice (GCP) guidelines and the World Medical Association’s Declaration of Helsinki.
