# Supplementary material for: Label-free classification of breast cancer subtypes in ex vivo human tissues using Raman spectroscopy and machine learning
Source: Sci Rep. 2026 Jun 11;16:26557. doi: 10.1038/s41598-026-54071-5 (PMC13503704; doi:10.1038/s41598-026-54071-5)
Supplement: Supplementary file 1 [file 41598_2026_54071_MOESM1_ESM.docx]

**SUPPLEMENTARY INFORMATION**

**Label-free classification of breast cancer subtypes in *ex vivo* human tissues using Raman spectroscopy and machine learning**

Ahmed Ezzat1,2,†, Zhiyu Zhu1,2,†, Alfie Roddan1,2, Anna Silvanto2, Yingwei Hou1,2, Nilanjan Mandal1,2, Jiacheng Xu1,2, Ziqi Zhang1,2, Mengyi Zhou1,2, Ara Darzi1,2, Simon Dryden2, Daniel R. Leff1,2,††,*, Alex J. Thompson1,2,††,*

1Hamlyn Centre, Institute of Global Health Innovation, Imperial College London, London, SW7 2AZ, UK

2Department of Surgery and Cancer, Imperial College London, London, SW7 2AZ, UK

†,†† These authors contributed equally.

* Corresponding authors: [d.leff@imperial.ac.uk](mailto:d.leff@imperial.ac.uk); [alex.thompson08@imperial.ac.uk](mailto:alex.thompson08@imperial.ac.uk)

**This PDF file includes:**

Supplementary Figures S1-S6

Supplementary Tables S1-S5

**Supplementary Figures**

**Fig. S1.** Pre-processing process and background signal analysis of Raman spectra. (a) Comparison of the original spectra, the smoothed processed spectra and the baseline corrected spectra, with the marked asterisks and triangles indicating peak positions. (b) Zoom-in to show the raw and smoothed signals in the 1800-2000 cm^-1^ region, demonstrating the effect of the smoothing.

**Fig. S2**. Two-dimensional PCA visualization of Raman spectral data. (a) Two-class PCA results (normal and cancer) in three sets of principal component pairs (PC1 vs. PC2, PC2 vs. PC3, and PC1 vs. PC3). (b) Four-class PCA results (normal, DCIS, ILC and IDC) in the three sets of principal component pairs.

**Fig. S3**. LDA visualization of Raman spectral data for four-class classification. The scatter plots show the distribution of normal, DCIS, ILC and IDC samples along the first three linear discriminants (LD1 vs. LD2, LD2 vs. LD3, and LD1 vs. LD3).

**Fig. S4**. LDA classification results with 10-fold cross-validation. (a) Confusion matrix for binary classification (cancer vs. normal). (b) Classification accuracy as a function of number of PCs included in classification model for the binary task. Three highlighted markers indicate best accuracy (red triangle, 50 PCs), accuracy at the number of PCs explaining 90% of the total variance (purple square, 75 PCs), and accuracy at the number of PCs explaining 95% of total variance (yellow diamond, 104 PCs). Inset plot shows detail of graph around the highlighted accuracies. (c) Confusion matrix for four-class classification. (d) Classification accuracy as a function of number of PCs included in classification model for the four-class task. Three highlighted markers indicate best accuracy (red triangle, 90 PCs), accuracy at 90% explained variance (purple square, 75 PCs), and accuracy at 95% explained variance (yellow diamond, 104 PCs). Inset plot shows detail of graph around the highlighted accuracies. Confusion matrices were calculated based on optimal model performance (using 50 PCs for two-class classification, 90 PCs for four-class classification).

**Fig. S5**. SVM classification results with 10-fold cross-validation. (a) Confusion matrix for binary classification (cancer vs. normal). (b) Classification accuracy as a function of number of PCs included in classification model for the binary task. Three highlighted markers indicate best accuracy (red triangle, 99 PCs), accuracy at the number of PCs explaining 90% of the total variance (purple square, 75 PCs), and accuracy at the number of PCs explaining 95% of total variance (yellow diamond, 104 PCs). Inset plot shows detail of graph around the highlighted accuracies. (c) Confusion matrix for four-class classification. (d) Classification accuracy as a function of number of PCs included in classification model for the four-class task. Three highlighted markers indicate best accuracy (red triangle, 21 PCs), accuracy at 90% explained variance (purple square, 75 PCs), and accuracy at 95% explained variance (yellow diamond, 104 PCs). Inset plot shows detail of graph around the highlighted accuracies. Confusion matrices were calculated based on optimal model performance (using 99 PCs for two-class classification, 21 PCs for four-class classification).

**Fig. S6**. Accuracy comparison between LDA and SVM for two-class and four-class classification tasks. Identical randomized data ordering and cross-validation splits were used for both classifiers to enable paired comparison. A total of 100 paired cross-validation results were collected for each task. Boxes represent the interquartile range with median values indicated by red lines. Whiskers extend to most extreme (i.e. highest and lowest) data points. Statistical significance between LDA and SVM was assessed using the Wilcoxon signed-rank test (*** p < 0.001).

**Supplementary Tables**

**Table S1.** Representative Raman band assignments and associated biochemical components in breast tissue.

| **Peak (**$\boldsymbol{cm}^{\boldsymbol{-1}}$**)** | **Associated Vibrational Mode** | **Associated biochemical component** |
| --- | --- | --- |
| 937 | C–C stretching (proline-rich collagen; protein backbone, α-helix)^1^ | Proteins |
| 1003 | Symmetric ring breathing (phenylalanine)^1^ | Proteins |
| 1080 | Skeletal C-C stretching (chain random)^2^ | Lipids |
| 1158 | C–C stretching (carotenoid-associated, e.g., β-carotene)^2^ | Lipids |
| 1294 | CH₂ twisting / wagging (lipid chains)^3^ | Lipids |
| 1436 | CH₂ scissors^4^ | Lipids |
| 1526 | C=C stretching (carotenoid-associated, e.g., β-carotene)5 | Lipids |
| 1662 | Amide I (C=O stretching)^6^ | Proteins |
| 1747 | C=O stretching (ester groups in lipids)^7^ | Lipids |

**Table S2**. LDA classification performance metrics for two- and four-class classification tasks, including sensitivity, specificity, precision, overall accuracy, and accuracy at the number of PCs explaining 90% and 95% of the total variance (based on 10-fold cross-validation). Metrics (except for 90/95% explained variance accuracy) were calculated based on optimal model performance (using 50 PCs for two-class classification, 90 PCs for four-class classification).

| **Classification Task** | **Classes** | **Sensitivity** | **Specificity** | **Precision** | **Total Accuracy** | **90/95% Explained Variance Accuracy** |
| --- | --- | --- | --- | --- | --- | --- |
| **Two-class** | **Cancer vs. Normal** | 87.07% | 88.70% | 95.28% | 87.5% | 85.96% /  84.87% |
| **Four-class** | **DCIS vs. Non-DCIS** | 82.35% | 84.76% | 73.98% | 75.35% | 73.95% /  72.55% |
|  | **IDC vs. Non-IDC** | 77.19% | 95.45% | 78.57% |  |  |
|  | **ILC vs. Non-ILC** | 51.16% | 95.90% | 75.86% |  |  |
|  | **Cancer vs. Normal** | 83.05% | 89.44% | 75.00% |  |  |

**Table S3**. SVM classification performance metrics for two- and four-class classification tasks, including sensitivity, specificity, precision, overall accuracy, and accuracy at the number of PCs explaining 90% and 95% of total variance (based on 10-fold cross-validation). Metrics (except for 90/95% explained variance accuracy) were calculated based on optimal model performance (using 99 PCs for two-class classification, 21 PCs for four-class classification).

| **Classification Task** | **Classes** | **Sensitivity** | **Specificity** | **Precision** | **Total Accuracy** | **90/95% Explained Variance**  **Accuracy** |
| --- | --- | --- | --- | --- | --- | --- |
| **Two-class** | **Cancer vs. Normal** | 97.41% | 91.53% | 96.79% | 95.79% | 95.16% /  95.32% |
| **Four-class** | **DCIS vs. Non-DCIS** | 94.12% | 83.33% | 74.82% | 83.00% | 80.97% /  79.88% |
|  | **IDC vs. Non-IDC** | 78.07% | 97.91% | 89.00% |  |  |
|  | **ILC vs. Non-ILC** | 62.79% | 97.66% | 87.10% |  |  |
|  | **Cancer vs. Normal** | 87.01% | 96.55% | 90.59% |  |  |

**Table S4.** LDA classification performance for subtype-specific binary tasks (Normal vs. each cancer subtype).

| **Class type** | **Accuracy** | **Sensitivity** | **Specificity** | **Precision** |
| --- | --- | --- | --- | --- |
| **Normal vs DCIS** | 97.25% | 96.38% | 98.31% | 98.61% |
| **Normal vs ILC** | 88.58% | 77.51% | 96.61% | 94.34% |
| **Normal vs IDC** | 95.21% | 87.72% | 100% | 100% |

**Table S5**. SVM classification performance for subtype-specific binary tasks (Normal vs. each cancer subtype).

| **Class type** | **Accuracy** | **Sensitivity** | **Specificity** | **Precision** |
| --- | --- | --- | --- | --- |
| **Normal vs DCIS** | 98.50% | 98.64% | 98.31% | 98.64% |
| **Normal vs ILC** | 96.06% | 99.22% | 93.79% | 92.09% |
| **Normal vs IDC** | 98.31% | 95.61% | 100% | 100% |

**Reference:**

[1] Huang, Z., McWilliams, A., Lui, H., McLean, D. I., Lam, S. & Zeng, H. Near-infrared Raman spectroscopy for optical diagnosis of lung cancer. *Int. J. Cancer* **107**, 1047–1052 (2003). <https://doi.org/10.1002/ijc.11500>
[2] Redd, D. C. B., Feng, Z. C., Yue, K. T. & Gansler, T. S. Raman spectroscopic characterization of human breast tissues: implications for breast cancer diagnosis. *Appl. Spectrosc.* **47**, 787–791 (1993). <https://doi.org/10.1366/0003702934067072>
[3] Kaminaka, S., Ito, T., Yamazaki, H., Kohoda, E. & Hamaguchi, H. Near-infrared multichannel Raman spectroscopy toward real-time in vivo cancer diagnosis. *J. Raman Spectrosc.* **33**, 498–502 (2002).
[4] Frank, C. J., McCreery, R. L. & Redd, D. C. B. Raman spectroscopy of normal and diseased human breast tissues. *Anal. Chem.* **67**, 777–783 (1995).
[5] Stone, N., Kendall, C., Smith, J., Crow, P. & Barr, H. Raman spectroscopy for identification of epithelial cancers. *Faraday Discuss.* **126**, 141–157 (2004). <https://doi.org/10.1039/B304992B>
[6] Frank, C. J., McCreery, R. L. & Redd, D. C. B. Raman spectroscopy of normal and diseased human breast tissues. *Anal. Chem.* **67**, 777–783 (1995). <https://doi.org/10.1021/ac00101a001>
[7] Lakshmi, R. J., Kartha, V. B., Murali Krishna, C., Solomon, J. G. R., Ullas, G. & Uma Devi, P. Tissue Raman spectroscopy for the study of radiation damage: brain irradiation of mice. *Radiat. Res.* **157**(2), 175–182 (2002). <https://doi.org/10.1667/0033-7587(2002)157%5B0175:TRSFTS%5D2.0.CO;2>
